# Fractal and Gray Level Cooccurrence Matrix Computational Analysis of Primary Osteosarcoma Magnetic Resonance Images Predicts the Chemotherapy Response

**DOI:** 10.3389/fonc.2017.00246

**Published:** 2017-10-19

**Authors:** Goran J. Djuričić, Marko Radulovic, Jelena P. Sopta, Marina Nikitović, Nebojša T. Milošević

**Affiliations:** ^1^Department of Diagnostic Imaging, University Children’s Hospital, University of Belgrade, Belgrade, Serbia; ^2^Institute of Oncology and Radiology of Serbia, Belgrade, Serbia; ^3^Medical Faculty, Institute of Pathology, University of Belgrade, Belgrade, Serbia; ^4^Medical Faculty, Department of Biophysics, University of Belgrade, Belgrade, Serbia

**Keywords:** chemotherapy, fractals, gray level cooccurrence matrix, image analysis, magnetic resonance imaging, osteosarcoma, space-filling ratio

## Abstract

The prediction of induction chemotherapy response at the time of diagnosis may improve outcomes in osteosarcoma by allowing for personalized tailoring of therapy. The aim of this study was thus to investigate the predictive potential of the so far unexploited computational analysis of osteosarcoma magnetic resonance (MR) images. Fractal and gray level cooccurrence matrix (GLCM) algorithms were employed in retrospective analysis of MR images of primary osteosarcoma localized in distal femur prior to the OsteoSa induction chemotherapy. The predicted and actual chemotherapy response outcomes were then compared by means of receiver operating characteristic (ROC) analysis and accuracy calculation. Dbin, Λ, and SCN were the standard fractal and GLCM features which significantly associated with the chemotherapy outcome, but only in one of the analyzed planes. Our newly developed normalized fractal dimension, called the space-filling ratio (SFR) exerted an independent and much better predictive value with the prediction significance accomplished in two of the three imaging planes, with accuracy of 82% and area under the ROC curve of 0.20 (95% confidence interval 0–0.41). In conclusion, SFR as the newly designed fractal coefficient provided superior predictive performance in comparison to standard image analysis features, presumably by compensating for the tumor size variation in MR images.

## Introduction

Primary osteosarcoma occurs most commonly in the second decade of life (1). In the prechemotherapy era, osteosarcoma was characterized by very poor survival rates of less than 20% over a 5-year period. An improvement of this rate to over 80% was observed in the 1970s and early 1980s upon emergence of the chemotherapy regimens.

Osteosarcoma is treated by a multimodal therapy, comprising a preoperative chemotherapy, surgical treatment, and postoperative chemotherapy. The problem with the current clinical treatment of osteosarcoma is that the patient survival rate attained in the 1980s remains unchanged (2). The major conceivable strategy for the improvement in outcomes is based on the introduction of personalized treatment tailoring. This is currently not possible because reliable predictors of induction chemotherapy response are not available, although there are several therapeutic options for osteosarcoma (2). The degree of tumor necrosis in response to chemotherapy by pathohistological analysis is the most reliable assessment of the chemotherapy response and prognosticator of disease outcome, but it is only available after chemotherapy completion (3). A less reliable but earlier chemotherapy response evaluation, after at least one cycle of chemotherapy, has become possible more recently by magnetic resonance imaging (MRI) and functional imaging methods (4). Yet, a prediction of chemotherapy response prior to chemotherapy start is the optimal goal for the treatment tailoring that could avoid metastasis development during long ineffective treatments.

The absence of chemotherapy predictors has been stimulating the predictive marker discovery research. The main emphasis has been on molecular biomarkers, including proteins (5) and mRNA (6) which remain in early experimental phases with uncertain prospects of their implementation.

The computational structural analysis of the tumor morphology thus evolves as a strategy complementary to the mainstream molecular analysis in search for the improved prediction of chemotherapy response. This approach mostly uses the fractal geometry which has been developed with an intent to resolve the shortcomings of traditional geometry in structural analysis of irregular natural objects (7). Although fractal analysis has been widely used in analysis of magnetic resonance (MR) images and considered as the next generation quantitative analysis tool (8), it has never been applied to a predictive purpose in osteosarcoma.

The aim of this study was to test whether the gray level cooccurrence matrix (GLCM) and monofractal computational analyses of routinely collected osteosarcoma MR images prior to chemotherapy might have any capability to predict the chemotherapy response.

## Materials and Methods

The study was approved by the Institutional Review Board (Belgrade University, School of Medicine, approval #29/VI-4) and conforms with The Code of Ethics of the World Medical Association (Declaration of Helsinki), printed in the *British Medical Journal* (18 July 1964) and its 7th revision in 2013. Patient data were received by the pathology unit in a deidentified and recoded form without direct or indirect identifiers that could enable reidentification. This retrospective study was performed on archived and unidentifiable images acquired as part of a routine care and not for research purpose and for these reasons it was granted a waiver of patient consent by the IRB in adherence with the 2012 Health Insurance Portability and Accountability Act.

### Patient Group

Cross-sectional study encompassed a group of patients suffering from primary sarcoma in distal femur, all diagnosed and treated by the National Sarcoma Consilium during the 5-year period (2010–2014) at the Institute for Oncology and Radiology. The inclusion criteria aimed to homogenize the tumor location, presence of metastasis and pathological fracture. Of the 60 patients, 32 had tumors in distant femur. Ten of these 32 were excluded based on pathological fractures or metastasis to obtain a final patient group of 22. Homogenization was needed in this initial study as osteosarcoma is a highly heterogeneous disease. The sample size calculation required 20 patients (9 poor responders and 11 good responders) for the area under the receiver operating characteristic (ROC) curve (AUC) effect size of 0.80 (MedCalc Software, Ostend, Belgium). Patients were subjected to OsteoSa MAP neoadjuvant therapeutic protocol (doxorubicin, cisplatin, methotrexate). MRI was performed for all patients, confirming the presence and extent of the tumor formation.

### Chemotherapy Response Evaluation

As the study was retrospective, the patient stratification into good and poor chemotherapy responder groups was done according to their actual response. Pathohistological evaluation of the achieved chemotherapeutical effect was performed on the tumor tissue obtained by the tumor-removal surgery and not by biopsy. This evaluation was based on pathohistological Huvos grading system which is still the most widely used and most reliable method for the assessment of response to therapy (4). Grading was performed by the expert pathologist (Jelena P. Sopta) with 21 years of experience. Patients with the level of tumor necrosis exceeding 90% were considered as “good responders,” while those with less than 90% of necrosis were considered as “poor responders.”

### Image Acquisition

Images were obtained by the diagnostic workstation (Siemens Magnetom Avanto Syngo MR B15, Siemens Healthcare, Erlangen, Germany) and exported in 1,920 × 1,080 pixel size by use of the Kodak Carestream PACS Client Suite version 10.2 software (Kodak, Rochester, NY, USA). Tumor tissue was imaged in the coronary, sagittal, and transversal planes. Six images were analyzed per patient, with two characteristic images for each of the three spatial planes, selected by the expert radiologist (Goran J. Djuričić). The final number of images was thus 6 × 22 = 132.

### Fractal Analysis

Box-counting measures fractal dimension of real objects by covering the object with rectangular coordinate grid of cell size *r* and counts the number of boxes where the cell size is expressed as the number of foreground pixels. The number of squares *N*(*r*) needed to cover the image is given by a power law:

(1) $$N(r)=\text{const}\cdot r^{-D},$$

where *D* is the box dimension, calculated as an absolute value of the slope of the log–log relationship between *N*(r) and *r* as previously described in detail (9).

Four morphometric parameters were obtained by the box-counting method. Fractal dimension (Dbin) and outline fractal dimension (Dout) were calculated by respective use of binary and outline images produced by ImageJ. Dbin estimates the distribution of black pixels in a binary image of a tumor, while Dout estimates the shape of a tumor.

The space-filling ratio (SFR) was calculated as the ratio of two binary box dimensions for each image. The first Dbin derived from image with the whole tumor area flood-filled with foreground pixels (Figure 2C), while the second was derived from the inverted version of the same image in which the whole tumor area was filled, this time with background pixels. Fractal dimension and lacunarity (Λ) were calculated by the FracLac plugin for Image J (7). Lacunarity estimates gaps in binary images as irregularity in pixel distribution (Figure 2B) and the translational and rotational invariance of an image.

### GLCM Analysis

Generally, texture features contain information about the spatial distribution of tonal variations within a group of pixels: homogeneity, gray-tone linear dependencies, contrast, boundaries, and the complexity of the image. GLCM is the most commonly used type of texture analysis. It is defined as a two-dimensional histogram of gray levels for pairs of pixels, which are separated by a fixed spatial relationship (10). The number of rows and columns in the matrix is equal to the number of gray levels (*G*) in the image. The matrix element *P*(*i, j* | Δ*x*, Δ*y*) is the relative frequency of occurrence of two pixels with intensities *i, j*, separated by a pixel distance (Δ*x*, Δ*y*).

Five GLCM features were calculated: angular second moment (SASM), inverse difference moment (SIDM), contrast (SCN), correlation (SCR), and entropy (SE) by the following equations:

(2) $$S_{\text{ASM}}=\sum_{i=0}^{G-1}\sum_{j=0}^{G-1}{\left(P(i,j)\right)}^{2},$$

(3) $$S_{\text{IDM}}=\sum_{i=0}^{G-1}\sum_{j=0}^{G-1}\frac{1}{1+{\left(i-j\right)}^{2}}\cdot P(i,j),$$

(4) $$S_{\text{CN}}=\sum_{n=0}^{G-1}n^{2}\left(\sum_{i=1}^{G}\sum_{j=1}^{G}P(i,j)\right),n=\left|i-j\right|,$$

(5) $$S_{\text{CR}}=\sum_{i=0}^{G-1}\sum_{j=0}^{G-1}\frac{\left(i\cdot j)\cdot P(i,j)-(\mu_{x}\cdot \mu_{y}\right)}{\left(\sigma_{x}\cdot \sigma_{y}\right)},$$

(6) $$S_{E}=-\sum_{i=0}^{G-1}\sum_{j=0}^{G-1}P(i,j)\cdot \text{log}\left(P(i,j)\right),$$

where μ and σ are the mean and SDs of probabilities *P_x_* and *P_y_*.

While SASM (homogeneity) measures textural uniformity of the image, SIDM measures local image homogeneity as it assumes larger values for smaller gray tone differences in pair elements. Furthermore, SCN measures the spatial tonal frequency of an image as the difference between the highest and the lowest values of a contiguous set of pixels, while SCR is a measure of gray tone linear dependencies in the image. Finally, the SE estimates the amount of information that is needed for image compression, in other words, it measures disorder and complexity.

### Data Categorization and Statistical Analyses

The measured continuous values were categorized in order to enable allocation of patients into the good- and poor-responder groups. The best threshold values for each parameter in univariable analysis were calculated by X-tile 3.6.1 software, Yale University, New Haven, CT, USA (11), followed by a stepwise multivariable logistic regression analysis. Variables categorized by outcome were added to a full model using forward selection entry criterion of *P* < 0.10 in univariate analysis and removed using backward elimination according to a selection stay criterion of *P* < 0.05.

Areas under the receiver operating characteristic (ROC) curves were calculated as a quantitative measure of discrimination efficiency. AUCs were computed based on continuous feature values. Classification accuracy was used as the additional prognostic measure, indicating the percentage of times that the predicted and actual outcomes match (12). Accuracies were calculated by use of categorized values. The bootstrap random resampling technique was applied for bias correction. This procedure tests model stability and reliability by estimating the bias and then corrects bias by modification of the original AUC confidence intervals (95% CIs) as previously explained in detail (13). The bias is the difference between the calculated uncorrected 95% CI for AUC and their true values. The advantage of bootstrap over the split-sample cross-validation as another major internal validation method is that the entire dataset is used for model development. The SPSS software package v23 (IBM SPSS Statistics, Chicago, IL, USA) was employed for these statistical analyses.

## Results

The predictive value of monofractal and GLCM analyses for the primary osteosarcoma tumors was retrospectively evaluated in the patient group which was preoperatively treated with the OsteoSa MAP therapeutic protocol. MR images were obtained before the chemotherapy application. The actual response of each patient to chemotherapy was determined by the pathohistological examination at the time of surgery. It was found that chemotherapy response predicted by SFR provided the best association with the actual chemotherapy response.

Several clinicopathological and demographic parameters were available, including gender, age, tumor resection margins, metastasis, pathological fracture, tumor surface area, and tumor volume (Table 1). ROC analysis based on the continuous numerical values did not indicate a significant predictive power for any of the clinicopathological parameters (not shown). However, the binary logistic regression based on the values categorized by the best threshold did indicate several significant clinicopathological predictors (Table 1).

**Table 1:** Patient characteristics.

Characteristics	*n*	*P*-value^a^^,^^b^	Hazard ratio^a^^,^^b^	95% CI^a^^,^^b^
**Age**				
< 20	18 (82%)			
> 20	4 (18%)	0.06	0.16	2.3 × 10^−10^–1.3
Median	13			
**Gender**				
Male	15 (68%)	0.05	0.20	1.9 × 10^−10^–1.2
Female	7 (32%)			
**Tumor volume (cm^3^)**				
Median	1,476	0.40	0.48	0.04–3.0
Range	85–10,590			
**Tumor surface (cm^2^)**				
Median	19.2	0.43	0.50	0.02–3.3
Range	3.2–57.7			
**Pattern on MRI**				
Concentric	19 (86%)	0.72	0.47	0.10–2.1
Longitudinal	3 (14%)			
**Histologic response**				
Good	12 (54%)	–	–	–
Poor	10 (46%)	–	–	–
**Pathologic subtype**				
Osteoblastic	10 (46%)	–	–	–
Chondroblastic	6 (27%)	–	–	–
Fibroblastic	4 (18%)	–	–	–
Other	2 (9%)	–	–	–
**Location**				
Distal femur	22 (100%)	–	–	–
**Metastatic disease**				
No	22 (100%)	–	–	–

*Major demographic, clinicopathological, and MRI characteristics of the patient group and their predictive evaluation by the binary logistic regression test*.

*^a^Binary logistic regression test, bootstrap corrected*.

*^b^Obtained with data categorized by an optimal threshold*.

*CI, confidence interval; n, number of patients*.

**Figure 1:**
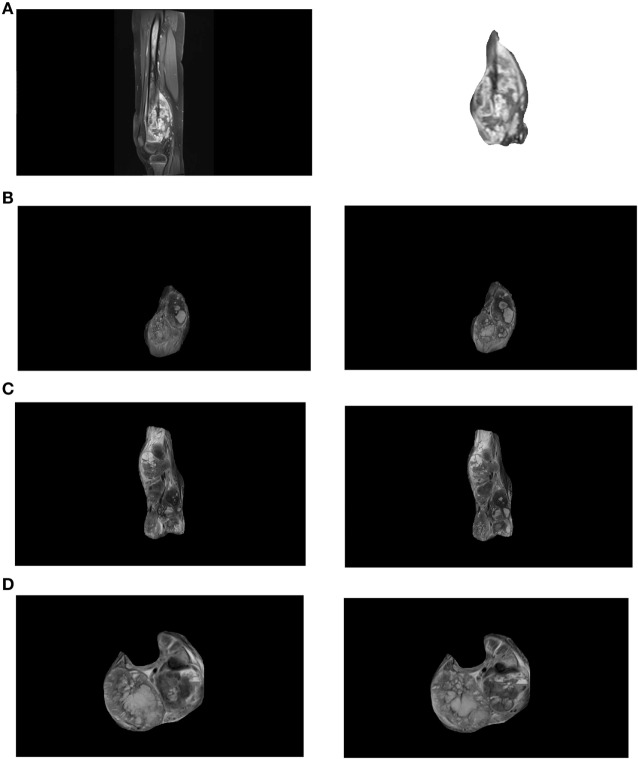
**(A)** An exemplary distal femur magnetic resonance (MR) image (left) and a region of interest showing the tumor (right). Examples of tumor images recorded in coronal **(B)**, sagittal **(C)**, and transversal **(D)** planes. For each patient two characteristic images in each plane [**(B–D)** left and right] were analyzed.

**Figure 2:**
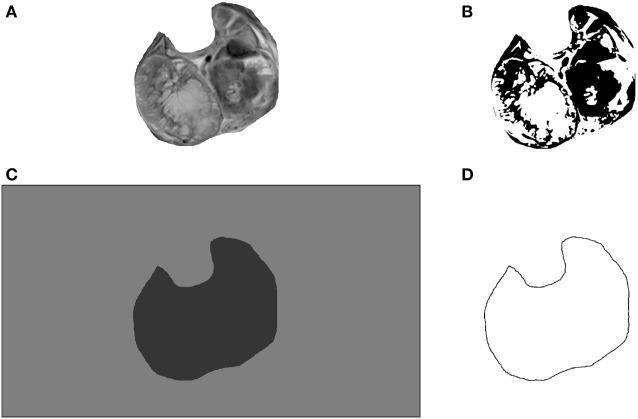
An example of image processing for computational image analysis: grayscale **(A)**, binarized **(B)**, binarized with filled region of interest (ROI) **(C)** and **(D)** binarized outline of the whole tumor area.

Images (Figure 1A left) were cropped by Image J software to isolate regions of interest (ROIs) according to borders of each individual tumor (Figure 1A right). The sizes of ROIs reflected the actual variation of tumor dimensions. Such type of image processing is commonly used to obtain the optimally comparable and relevant image details for both fractal and texture analyses. Examples of images in coronal, sagittal, and transversal planes are shown in Figures 1B–D, respectively. Each image was saved in grayscale (Figure 2A), converted from grayscale to binary (Figure 2B) with ROI flood-fill (Figure 2C) and outline versions (Figure 2D).

MR image analysis results are shown in Tables 2 and 3. SFR significantly associated with the chemotherapy outcome in the coronal and sagittal, but not in transversal plane (Table 2). Λ and SCN were significant predictors in the coronal plane and Dbin in the sagittal section (Tables 2 and 3). When data from all three planes were averaged for each feature, only SFR was significant (Tables 2 and 3).

**Table 2:** The prognostic significance of the examined box-count features.^a^^,^^b^

*D*_bin_	*D*_out_	SF_R_	Λ
**Coronal section**			
0.51	0.74	**0.04**	**0.02**
0.42; 0.16–0.68	0.54; 0.29–0.79	**0.30**; 0.08–0.53	**0.21**; 0.01–0.40
**Sagittal section**			
**0.05**	0.92	**0.03**	0.67
**0.30**; 0.07–0.49	0.49; 0.24–0.74	**0.31**; 0.08–0.47	0.45; 0.19–0.71
**Transversal section**			
0.43	1.0	0.20	0.51
0.40; 0.15–0.65	0.50; 0.24–0.76	0.34; 0.11–0.57	0.58; 0.34–0.83
**Average**			
0.08	0.95	**0.02**	0.32
0.30; 0.06–0.51	0.49; 0.23–0.75	**0.20**; 0–0.41	0.38; 0.14–0.62

*ROC analysis was used for evaluation of predictive significance. Each result indicates a P-value followed by AUC and its 95% confidence interval*.

*^a^AUC values and 95% CI are bootstrap corrected*.

*^b^Obtained with continuous data*.

*Dbin, fractal dimension obtained on binarized images; Dout, fractal dimension obtained on binarized and outlined images; SFR, ratio of the Dbins obtained on images in which tumor ROI was filled with foreground and background pixels; Λ, lacunarity*.

*Boldface values indicate significance with p < 0.05*.

**Table 3:** The prognostic significance of the examined GLCM features.^a^^,^^b^

*S*_ASM_	*S*_IDM_	*S*_CN_	*S*_CR_	*S*_E_
**Coronal section**				
0.79	0.69	**0.04**	0.32	0.90
0.53; 0.28–0.79	0.45; 0.20–0.70	**0.73**; 0.52–0.95	0.38; 0.13–0.62	0.52; 0.27–0.77
**Sagittal section**				
0.39	1.0	0.29	0.58	0.69
0.61; 0.37–0.85	0.50; 0.25–0.75	0.63; 0.39–0.87	0.43; 0.17–0.69	0.45; 0.20–0.70
**Transversal section**				
*0.25*	0.74	0.95	0.19	0.64
0.65; 0.40–0.89	0.46; 0.21–0.71	0.49; 0.23–0.75	0.67; 0.43–0.90	0.44; 0.19–0.69
**Average**				
0.24	0.69	0.24	0.84	0.64
0.65; 0.41–89	0.45; 0.20–0.70	0.65; 0.42–0.88	0.53; 0.27–0.78	0.44; 0.19–0.69

*ROC analysis was used for evaluation of predictive significance. Each result indicates a P-value followed by AUC and its 95% confidence interval*.

*^a^AUC values and 95% CI are bootstrap corrected*.

*^b^Obtained with continuous data*.

*SASM, angular second moment; SIDM, inverse difference moment; SCN, contrast; SCR, correlation; SE, entropy*.

*Boldface values indicate significance with p < 0.05*.

The calculated accuracies for the significant chemotherapy predictors in Tables 2 and 3 were: SFR COR 68%, Λ COR 77%, Dbin SAG 77%, SFR SAG 70%, SCN CORO 77%, and SFR AVER 82%.

Correlation cross-analysis of the clinicopathological parameters and texture features has revealed the significant correlation between SFR TRANSV with the tumor area (Spearman coefficient = 0.64) and SFR TRANSV with the tumor volume (Spearman coefficient = 0.57).

**Figure 3:**
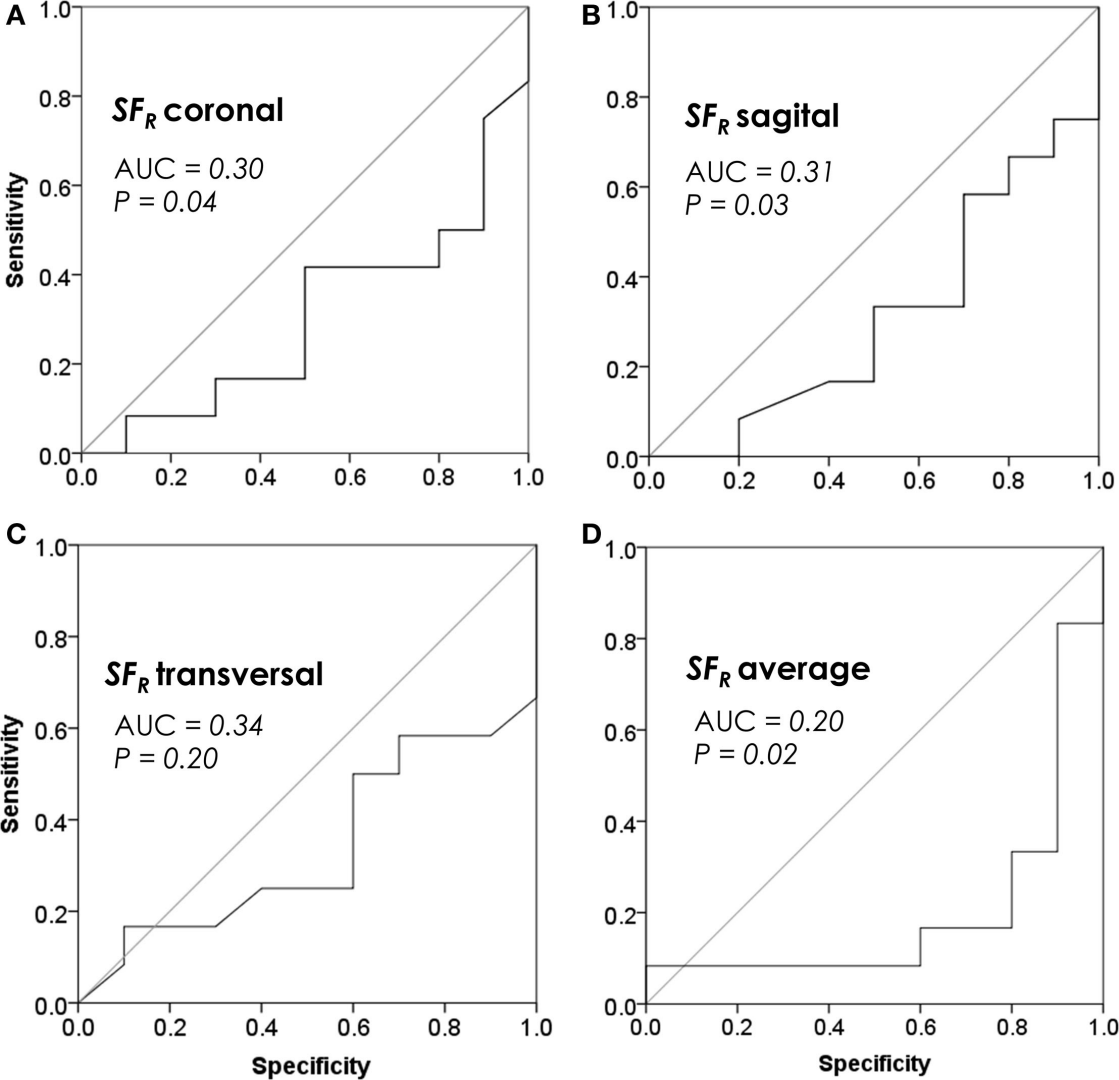
Prognostic performance of space-filling ratio (SFR) by receiver operating characteristic (ROC) analysis. **(A)** SFR in the coronal section, **(B)** SFR in the sagittal section, **(C)** SFR in the transversal section, and **(D)** SFR averaged among all three sections. Plots reveal discrimination efficiencies of SFR continuous values, prior to their categorization.

The multivariate analysis was performed to capture the redundancy between features averaged across the three sections (Table 4). SFR AVER was thereby indicated as an independent feature able to predict chemotherapy response after adjustment with all significant clinicopathological and texture chemotherapy predictors (Table 4). A detailed analysis of the SFr discrimination efficiency is presented in Figure 3.

**Table 4:** Multivariate binary logistic regression analysis of the chemotherapy response.^a^

	Coefficient	*P*-value^b^
Age	17.864	0.028
SFr	−38.292	0.001
Λ	−55.947	0.004
*S*_ASM_	57.072	0.001
*S*_IDM_	−37.338	0.001

*Multivariate analysis was performed by inclusion of all significant predictors to capture their predictive redundancy*.

*^a^Average values of the three planes*.

*^b^Binary logistic regression, bootstrap corrected*.

*Λ, lacunarity; SASM, angular second moment; SIDM, inverse difference moment; SFr, space-filling ratio*.

## Discussion

A prediction of chemotherapy response at the time of diagnosis may prove beneficial for osteosarcoma patients. Their initial treatment consists of either induction chemotherapy or amputation, mainly depending on the estimated size of surgical margins. Due to a lack of reliable chemotherapy outcome predictors at the time of diagnosis, chemoresistance remains the main limit of the clinical effectiveness in osteosarcoma (2). Primary tumors are the obvious source of predictive clues for chemotherapy response (9). The present study therefore investigated the chemotherapy response prediction of the computational morphologic evaluation in primary tumors. The neoadjuvant chemotherapy model used in this study is currently accepted as an optimal approach for assessment of the *in vivo* neoadjuvant chemotherapy response because a tumor remains *in situ* all until surgery (14).

Interestingly, the performance of image analysis was not symmetrical among the imaging planes. More specifically, three features exerted predictive significance in the coronal section, two in the sagittal section and none in the transversal section. Although several standard GLCM and fractal features, Dbin, Λ, and SCN, significantly associated with chemotherapy outcome, this was evident in only one of the three examined planes.

In an attempt to accomplish an improvement in prognostic value, we designed SFR as a normalized version of Dbin. This SFR compensated for the tumor size variability relative to background within MR images. Such effort has indeed resulted in a much more consistent predictive performance as SFR significantly associated with the chemotherapy response in two planes while it also trended toward significance in the remaining third plane (*P* = 0.20). SFR averaged among the three planes has achieved an AUC of 0.20, generally regarded as an excellent discrimination performance.

Fractal and GLCM image analyses have been widely used (15), but generally underexploited in MRI and never applied to osteosarcoma MRI. We believe that this has been mainly due to a problematic difference in tumor sizes relative to the whole image which needs to be compensated in order to obtain meaningful results. The novel SFR parameter thus presents a genuine innovation in fractal analysis and a potential breakthrough in computational MR image analysis, because tumor size variation is a general occurrence in MR images.

The inherent limitation of the present study is in the relatively small number of 22 patients. Therefore, additional studies in an extended patient group and in external groups would be needed to further characterize the predictive clinical validity of SFR. But even in this study the number of examined tumors derived from a larger group of 60 patients. This group was condensed by selection of highly homogeneous 22 patients in order to most reliably evaluate the predictive potential of fractal and GLCM analysis without influences from heterogeneities associated with the type of bone, presence of pathological fracture or metastasis. Besides the high homogeneity of the analyzed tumors, reliability of the obtained results is also based on the bootstrap resampling for internal validation of data which corrects the optimistic bias when the sample size is small (13). Furthermore, the used predictive evaluation was very stringent because AUC calculation was based on continuous values, thus avoiding the introduction of optimistic bias by the common categorization based on optimal threshold.

There have been no prior comparable studies specifically implemented to achieve a prediction of osteosarcoma chemotherapy response by fractal or GLCM MR image analysis. A single predictive study employing fractal MR image analysis was performed on breast tumors (16). A significant prognostic value was thereby achieved, but the quantitative association measures such as accuracy and AUC have not been calculated, thus limiting any direct comparison with our current results. Another study was focused on osteosarcoma chemotherapy response prediction but was restricted to molecular markers (6) and also did not provide either the predictive accuracy or AUC. Previous efforts in prediction of chemotherapy response that are comparable to our study (with predictions obtained before the chemotherapy onset) could achieve an accuracy of 78% by the average tumor area (17). This evaluation was performed on data categorized by the optimal threshold, a method known to introduce an optimistic bias. In our current study, tumor surface area did not deliver prognostic significance but the fractal SFr parameter achieved 82% accuracy by its continuous values. Others report the maximal accuracy of 59% (18) and AUC of 0.57 (4) by functional imaging prior to chemotherapy administration. Metabolic changes calculated by subtraction of values before and after chemotherapy treatment in the same study, included the postchemotherapy information and thus associated with an osteosarcoma chemotherapy response by a much improved AUC of 0.89 (4). Evidently, this parameter was not predictive as it made use of tumor measurements after the chemotherapy treatment. Our study implemented a fully predictive model since we only used MRI images before the start of chemotherapy to predict the future response to chemotherapy by the AUC of 0.80 and accuracy of 82%, achieved by SFr.

The clinical significance of chemotherapy response prediction at the time of diagnosis is based on the fact that tumor response to induction chemotherapy exerts a major influence on the disease outcome. Such chemotherapy is prescribed instead of an amputation in cases when wide surgical margins are achievable. The described method for prediction of chemotherapy resistance may thus be most applicable to patients with borderline surgical margins. An early identification of poor responders could thus prolong survival by directing to alternative treatments such as experimental protocols (clinical trials), an amputation or surgery.

The observed predictive power of MR image analysis could be explained by the unidentified tumor morphological qualities that are characteristic for either chemotherapy sensitivity or resistance. SFr is a derivative of fractal dimension based on pixel-level statistics and thus rather abstract. It was higher in poor responders (0.94 ± 0.01) in comparison to good responders (0.91 ± 0.02). With fractal dimension interpreted as a measure of complexity, it follows that increased complexity of a tumor was an indication of its chemotherapy-resistance.

In conclusion, the major novelty aspect of this study is the SFR fractal parameter designed to compensate for the variability in tumor sizes. The theoretical advantage of this parameter was here supported experimentally by its superior and independent predictive performance in comparison to standard fractal and GLCM features in analysis of MR images. Furthermore, SFR outperformed the previously reported volumetric and functional imaging parameters acquired before the onset of chemotherapy. An early prediction of the chemotherapy response long before surgery might benefit the patients by enabling the personalized tailoring of treatments.

## Ethics Statement

The study was approved by the Institutional Review Board (Belgrade University, School of Medicine, approval #29/VI-4) and conforms with The Code of Ethics of the World Medical Association (Declaration of Helsinki), printed in the *British Medical Journal* (July 18, 1964) and its 7th revision in 2013.

## Author Contributions

GD: MRI imaging, initial image formatting, agreement to be accountable for all aspects of the work, revisiting, and final approval. MR: statistics, interpretation of results, drafting the work, and agreement to be accountable for all aspects of the work. JS: study design, drafting the work, agreement to be accountable for all aspects of the work, and final approval. MN: study design, input on the clinical value of data, agreement to be accountable for all aspects of the work, and final approval. NM: design of the SFR fractal feature, fractal and GLCM image analysis, agreement to be accountable for all aspects of the work, revisiting, and final approval.

## Conflict of Interest Statement

The authors declare that the research was conducted in the absence of any commercial or financial relationships that could be construed as a potential conflict of interest.
